# Antibiotics or No Antibiotics, That Is the Question: An Update on Efficient and Effective Use of Antibiotics in Dental Practice

**DOI:** 10.3390/antibiotics10050550

**Published:** 2021-05-09

**Authors:** Alessio Buonavoglia, Patrizia Leone, Antonio Giovanni Solimando, Rossella Fasano, Eleonora Malerba, Marcella Prete, Marialaura Corrente, Carlo Prati, Angelo Vacca, Vito Racanelli

**Affiliations:** 1 Unit of Internal Medicine “Guido Baccelli”, Department of Biomedical Sciences and Human Oncology, University of Bari Medical School, 70124 Bari, Italy; alessio.buonavoglia@uniba.it (A.B.); patrizia.leone@uniba.it (P.L.); antonio.solimando@uniba.it (A.G.S.); rossella.fasano@uniba.it (R.F.); eleonora.malerba@uniba.it (E.M.); marcella.prete@uniba.it (M.P.); angelo.vacca@uniba.it (A.V.); 2 Department of Veterinary Medicine, University of Bari, 70010 Bari, Italy; marialaura.corrente@uniba.it; 3 Endodontic Clinical Section, Department of Biomedical and NeuroMotor Sciences, Dental School, University of Bologna, 40125 Bologna, Italy; carlo.prati@unibo.it

**Keywords:** antimicrobial resistance (AMR), antibiotics, oral infections, dental medicine

## Abstract

The antimicrobial resistance (AMR) phenomenon is an emerging global problem and is induced by overuse and misuse of antibiotics in medical practice. In total, 10% of antibiotic prescriptions are from dentists, usually to manage oro-dental pains and avoid postsurgical complications. Recent research and clinical evaluations highlight new therapeutical approaches with a reduction in dosages and number of antibiotic prescriptions and recommend focusing on an accurate diagnosis and improvement of oral health before dental treatments and in patients’ daily lives. In this article, the most common clinical and operative situations in dental practice, such as endodontics, management of acute alveolar abscesses, extractive oral surgery, parodontology and implantology, are recognized and summarized, suggesting possible guidelines to reduce antibiotic prescription and consumption, maintaining high success rates and low complications rates. Additionally, the categories of patients requiring antibiotic administration for pre-existing conditions are recapitulated. To reduce AMR threat, it is important to establish protocols for treatment with antibiotics, to be used only in specific situations. Recent reviews demonstrate that, in dentistry, it is possible to minimize the use of antibiotics, thoroughly assessing patient’s conditions and type of intervention, thus improving their efficacy and reducing the adverse effects and enhancing the modern concept of personalized medicine.

## 1. Introduction

The discovery of antibiotics has been one of the greatest advances in medical history, becoming an important instrument for treatment of infectious diseases and life-threatening postsurgical infections. However, over the past decades, an overuse and misuse of antibiotics has been observed, indiscriminately using these molecules to improve success rates of surgical techniques, heal suspected bacterial infections and prevent claims of negligence. These approaches, generally without scientific evidence, were integrated into common practice of clinicians, and patients used antibiotics as a sort of “drug of fear” or “*panacea*”.

The usage of antibiotics should be strictly linked to evidence-based efficacy to reduce economical costs and potential adverse effects. Furthermore the use of antibiotics can pose the risks of spreading mutant strains that show antibiotic resistance [1].

Antimicrobial resistance (AMR) has important consequences in management of life-threatening infections, therapy of immunocompromised patients and hospitalized intensive care patients. It is estimated that, every year, AMR causes 700,000 deaths globally. In Europe, it is estimated that there are 4 million antibiotic-resistant infections with 37,000 deaths and an economic impact of EUR 1.5 billion a year. In the USA, 2 million antibiotic-resistant infections with 50,000 deaths along with a healthcare expenditure of USD 20 billion a year have been reported. Moreover, it has been estimated that deaths caused by AMR bacteria could reach 10 million per year in 2050 worldwide [2].

The AMR phenomenon and attention to multidrug-resistant bacteria have had a sudden comeback with the COVID-19 emergency because secondary bacterial infections requiring antibiotic therapy are common complications of viral respiratory infections. Moreover, patients affected by COVID-19 are treated with empiric antibiotic prophylaxis such as macrolides [3].

The importance of a rational prescription of antibiotics is crucial in order to contrast the AMR phenomenon with its economic implications and negative impacts on healthcare systems. In several cases, antibiotics can be avoided without clinical complications. Additionally, antibiotics can cause adverse effects such as toxicity, allergic reactions and negative interactions with other drugs.

The important role of antibiotic prescriptions and subsequent risk of AMR are enhanced by dentists and oral surgeons. It has been estimated that 10% of antibiotic prescriptions are related to dental practice and that their use is not always linked to real indications and needs [4,5]. A default antibiotic prescription during dental procedures is still commonly recommended based on the old theory of “oral focal sepsis”, which indicates oral infections and/or oral surgical interventions as a possible source of bacteremia and subsequent spread of bacteria to other organs [6]. Indeed, antibiotics are prescribed with the aim of improving the success rates of surgical interventions, reducing complications and symptoms, and managing oral infections. Dentists used to prescribe antibiotics in cases of oro-dental pains and for postsurgical prophylaxis to avoid complications. However, these evaluations seem to be related to personal experience and old evidence rather than real indications. Furthermore, guidelines for a prudent usage of antibiotics are not adequately shared among practitioners. Several postsurgical complications or oro-facial pains are not necessarily linked to infection and antibiotics are prescribed with reduced dosages and reduced timing of administration, sometimes under patients’ requests.

Another critical point in dentistry is the empirical prescription of antibiotics without identification and cultivation of pathogens and in vitro susceptibility tests.

The aim of this review is to pinpoint and resume the most recent discoveries on antibiotics in oral surgery and dentistry, focusing on real clinical needs with the possibility to reduce antibiotic consumption and related AMR risks.

## 2. Types of Antibiotics and Administration Protocols

Antibiotics can be used for therapy or prophylaxis: antibiotic therapy (AT) assumes the presence of bacterial infection and the duration of treatment has to be prolonged even in absence of clinical of signs and symptoms of infection. Antibiotic prophylaxis (AP) is usually recommended in absence of an infection for preventing the risk of a local or disseminated infection. Disseminated infections may originate from bacterial penetration through incisions or wounds to the bloodstream, causing an infection in a distant organ or sepsis. Some categories of patients are more susceptible to this eventuality and AP is highly recommended for them. AP or AT protocols can include the use of antibiotics before, during and after surgery, based on specific treatment schemes linked to factors related to patient’s systemic health status and type of surgery [7,8].

Surgical techniques can be classified depending on the risk of the infections in clean surgery, clean-contaminated surgery, contaminated surgery and infected surgery [9]. Oro-maxillofacial surgery is a clean-contaminated surgery, as inflammation and contamination are not present but the risk of infection due to bacteria normally present on these tissues has to be considered. Consequently, perioperative AP has a real effectiveness in preventing transient bacteremia [10].

AP must be considered as a possible choice related to a specific case and a specific patient in order to modulate bacterial load and potential inflammation and promote an atraumatic surgical technique [11]. On the other hand, treatment of suppurative oro-facial infections must be classified as septic surgeries, although in this case the use of antibiotics as AT can be reduced and modulated according to specific clinical situations [12].

Short AP protocols are applied to reach empirically antibiotic concentrations that are 3–4-fold higher than the minimal inhibitory concentrations (MICs), only during the intrasurgical period, to prevent and contrast bacteremia or local bacterial contaminations, and they are not generally associated with the risk for postoperative AMR infections [13]. When AP is adopted to reduce bacteremia, surveys carried out in Europe show that amoxicilline is the most used antibiotic in preoperative protocols due to its efficacy, better absorption and lower risk of side effects. AP needs to be repeated at an interval of at least two weeks for multiple invasive procedures [14,15].

The oral microbiome is incredibly complex, with 200 predominant bacterial species and 700 predominant taxa, with various interactions between different bacterial species [16]. For this reason, the use of antibiotics in dentistry is generally based on empirical therapies and first choice antibiotics used in dentistry are usually broad-spectrum molecules [12], such as betalactams (amoxicillin alone or in combination with clavunalate, cephalosporins) and semisynthetic macrolides (claritromycine and azytromicyne) [17] (Table 1 and Table 2).

Amoxicillin is commonly used as the preferred first-line treatment, especially in AP, due to its moderate spectrum, good biodisponibility and, when taken per os, high plasmatic concentrations and relatively low adverse effects. Amoxicillin’s β -lactam ring binds and inactivates penicillin-binding protein (PBP) 1A, an enzyme essential for bacterial cell wall synthesis, inducing cell lysis and death [18]. The combination with clavulanate extends the spectrum, including all beta-lactamase-producing bacteria and alpha haemolytic oral viridans, *Streptococcus* and *Staphylococcus aureus*. Despite also possessing a β-lactam ring, clavulanic acid has little efficacy as an antibiotic and works as a “suicide inhibitor”, irreversibly binding to a serine residue in the active site of the beta-lactamase and preventing enzymatic hydrolysis of amoxicillin and other penicillins.

The amoxicillin–clavulanic acid combination is commonly used at a 7:1 ratio (875 mg amoxicillin/125 mg clavulanic acid) to avoid clavulanic acid-related toxicity—i.e., diarrhoea and gastrointestinal side effects [14,15,19].

Cephalosporins (such as ceftriaxone, cefotaxime, ceftazidima, cefepim) can be used as second choice for treating severe infections. Ceftriaxone must be administered with intramuscular injection and this can be an advantage for patients with vomit or gastrointestinal dysfunction [17].

Macrolides are effective against a variety of aerobic and anaerobic Gram-positive and Gram-negative bacteria, with a typical usage in patients with betalactams allergy history. It is important to mention that azithromycin and clarithromycin may prolong QT intervals, which increase the risk of sudden cardiac death due to torsades de pointes. The advantages of aziythromycin usage are the reduced daily dosage and the reduced therapy durations (500 mg once a day for 3 days) compared with clarithromycin (500 mg twice a day for 7 days) [20].

Clindamycin is another antibiotic used in patients allergic for betalactams. It is effective against most Gram-positive and Gram-negative aerobes and anaerobes with a good distribution in most body tissues and a bone concentration approximating to that in the plasma. Clindamycin should be carefully employed in patients with positive anamnesis of enteritis due to its gastrointestinal side effects. The adult oral dosage is 300 mg every 6 h [12,15].

Another antibiotic widely used for pharmacological treatment of periodontal diseases is *metronidazole*, due to its efficacy against anaerobes and its capacity to reach good concentrations in saliva and tissues, with an adult oral dosage of 500 mg three times daily for 3–7 days. Metronidazole can be used alone as alternative to betalactams or in combination with betalactams [21]. The combination (500 mg amoxicillin + 500 mg metronidazole three times daily for 3–7 days) was initially proposed in association with nonsurgical treatment for aggressive and refractory periodontitis, and recently used also to treat patients affected or at risk of developing medical-related osteonecrosis of the jaws after invasive dental procedures [21].

## 3. Antibiotic Prophylaxis for Dental Procedures in Patients at Risk for Infection

**Figure 1:**
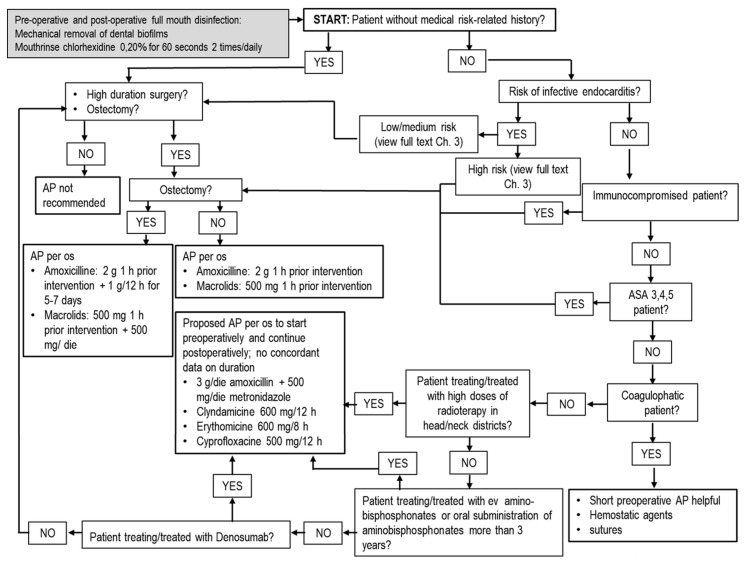
Flowchart for tooth extraction/resective surgical periodontology/mucogingival surgical periodontology in adult patients. In children, posology adjustments must be used as described in Table 1 and Table 2. Abbreviations: g = grams; mg = milligrams; h = hours; die = daily dosage; per os = oral administration; ASA = American Society of Anesthesiology; AP = Antibiotic prophylaxis.

**Figure 2:**
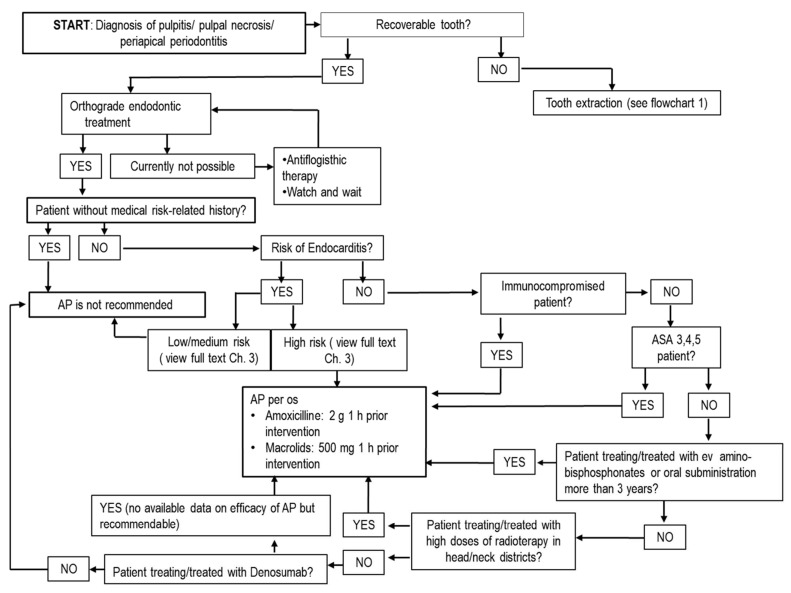
Flowchart for treatment of endodontic diseases in adult patients. In children, posology adjustments must be used, as described in Table 1 and Table 2. Abbreviations: g = grams; mg = milligrams; h = hours; per os = oral administration; ev = endovenous administration; ASA = American Society of Anesthesiology; AP = Antibiotic prophylaxis.

Invasive dental procedures are defined as techniques that require manipulation of gingival/periapical regions of teeth with/without perforation of alveolar mucosa. This includes oral surgery but also periodontal nonsurgical techniques (scaling, root planing), orthograde endodontics and subgingival dental preparations in fixed prosthesis. Bacteremia may occur as consequence of invasive procedures, at different rates—for instance, it has been reported in 18–85% of cases of tooth extraction and 60–90% of cases of periodontal surgery [14]. For nonsurgical periodontal treatments, such as scaling and root planing, the prevalence of bacteremia ranges from 13% to 90%, whereas it ranges from 0% to 42% for root canal treatment [14]. The risk of bacteremia in implantology is assessed at 7% [14]. Orthodontic treatment, conservative treatments and all noninvasive dental procedures in general are considered relatively safe, with only a remote possibility of inducing bacteremia [14]. Recently, it has been reported that bacteremia is not only closely related to surgical or invasive procedures, but it may also be linked to routine daily activities such as tooth brushing [22]. In this case, the duration and magnitude of bacteremia is not the same as after dental extractions, but the major cumulative exposition rate poses a similar risk, with high bacterial load in comparison to invasive procedures. This new research emphasizes the need for an improvement in oral hygiene and gingival health as protective factors against the risk of bacteremia linked to tooth brushing and as an alternative to the use of antibiotics [23]. Accordingly, antibiotics should be used only when necessary and in selected clinical situations for healthy patients. Prescribing schemes depends on type of diagnosis, type of treatment and type of patient (Table 1 and Table 2) [24]. Bacteremia occurs immediately after invasive dental procedures and decreases over time. In general, bacteria are cleared from the bloodstream by the host defense system from a few minutes to 1 h after dental procedure with no potential risks [22], but it can persist in medically compromised patients [22,23]. The category of medically compromised patients include two different groups with medical risk-related histories (Table 3) for whom AP may be of benefit and is recommended (Figure 1 and Figure 2). The first group is composed of patients at high risk of infective endocarditis (IE). In 2017, the American Heart Association (AHA) and American College of Cardiology (ACC) included patients with total/partial prosthetic cardiac valves in this category, including transcatheter-implanted prostheses and homografts and prosthetic material used for cardiac valve repair such as annuloplasty rings and chords, previous IE, unrepaired cyanotic congenital heart diseases (CHDs) such as tetralogy of Fallot, cardiac transplant with valve regurgitation due to a structurally abnormal valve and repaired CHD with residual shunts or valvular regurgitation at the site of or adjacent to the site of a prosthetic patch or prosthetic device [25]. For repaired CHD, AP is recommended during the first 6 months from cardio-surgery when prosthetic material was used for repair or a valvular regurgitation is near the prosthetic device. For these patients, the risk is related to bacteremia that, concomitant to other risk factors (prosthetic heart valve, congenital heart and valvular diseases and dysfunctions, previous IE), could cause a bacterial seed on endocardium and valves developing IE [25]. The incidence of IE in the background population is reported at around 6.0/100,000 person-year (PY), while in patients with prior IE (including patients who had valve surgery during the first IE hospitalization) is 16.1/1000 PY, 6.0/1000 PY for patients with prosthetic heart valves, and 1.5/1000 PY for patients with complex CHD [26]. The cumulative risk of IE for patients with previous IE was evaluated as 7.3% and 8.8% at 5 and 10 years, respectively. Patients who underwent valve surgery for first-time IE had incidences of new IE at 5 and 10 years of 6.7% and 8.7%. For patients with a prosthetic cardiac valve, the cumulative risks of IE were 2.8% and 4.5% at 5 and 10 years, and for patients with CHD, the cumulative risks were 0.9% and 1.3% at 5 and 10 years, respectively [26].

Several studies showed that “oral viridans group streptococci” (VGS) bacteremia and subsequent IE are presumably developed as a consequence of invasive dental procedures (reviewed in [25]). Accordingly, AP for the prevention of bacterial endocarditis secondary to dental procedures has been strongly recommended since 1955 [27]. Nevertheless, in recent years, a poor correlation between oral bacteremia and IE has been demonstrated [28] and the clinical effectiveness of AP used for dental procedures to prevent IE has to be proven [29,30]. Moreover, transient bacteremia can be also determined by tooth brushing with a major cumulative exposure risk with respect to invasive dental procedures [22]. Furthermore, there is a large number of case reports describing IE that developed many months after dental procedures with no detection of oral streptococci in endocardium lesions (reviewed in [31]). In 2008, on the basis of these evaluations, the National Institute for Health and Care Excellence (NICE) of the UK recommended that antibiotics should not be given routinely for IE prevention in the case of dental procedures to adults and children at risk of IE [32]. To support the NICE guidelines, retrospective studies reported no evidence of incidence of IE associated specifically with the date of withdrawal of dental AP (reviewed in [28]). In addition, the AHA, the American College of Cardiology (ACC) and the European Society for Cardiology (ESC) recommended restrictions of AP for patients at highest risk of IE. Patients with pre-existing native valve disease (including common conditions such as mitral valve prolapse, calcific aortic stenosis, bicuspid aortic valve) or rheumatic heart disease are considered to be at intermediate or low risk and AP is not strictly indicated [25]. For IE prevention, dental practitioners should follow AHA/ACC guidelines, prescribing AP for high-risk cardiac patients and emphasizing the importance of optimal oral health to reduce the incidence of bacteremia caused by activities of daily living, such as chewing, brushing, flossing. The suggested scheme is generally a short preoperative AP, eventually prolonged for other factors related to intervention [25]. If AP is inadvertently not carried out before the procedure, it may be administered up to 2 h after the intervention. Due to the nature of the pharmacokinetics of the AP regimen, a single loading dose is given in order to cover the period of potential bacteremia produced by a single procedure, so AP should be repeated prior to a hypothetical second appointment in the following day. In patients who require prophylaxis but are already taking antibiotics for another condition, it is recommended to select a different class of antibiotics from those the patient is already taking.

The second group includes immunocompromised patients, a category that is less capable of managing infections, with potential risks for a transient bacteremia or a postsurgical infection. Immunocompromization can be genetic or acquired (leukemia, AIDS, end-line nephropathies, uncontrolled diabetes, dialysis, immunosuppressive therapies or chemotherapies) and is characterized by leukopenia (<3500 u/mm^3^) or low seral levels of immunoglobulins (<2 g/L) [33].

Other risk categories are patients classified by the American Society of Anesthesiology (ASA) as grades 3 to 5. ASA 3 identifies patients with severe systemic diseases determining functional limitations of organs including (but not limited to) poorly controlled diabetes, renal failure requiring dialysis, active hepatitis, a healed myocardial infarction (MI) of more than 6 months, or patients who have undergone coronary artery bypass surgery (CABG), valve replacement, angioplasty and pacemaker or internal cardiac defibrillator implantation. In ASA 3, there are also patients with moderate reduction in the ejection fraction and patients with respiratory dysfunctions, such as bronchospastic disease with intermittent exacerbation. ASA 4 identifies patients with severe systemic disease, which is a constant threat to life. In this category, there are patients with unstable angina, poorly controlled chronic obstructive pulmonary diseases, symptomatic congestive heart failures (severe valve dysfunction, severe reduction in ejection fraction), recent (less than six months ago) myocardial infarction or stroke. ASA 5 patients are generally not included in routine dental practice and include moribund patients who are not expected to survive beyond the next 24 h without surgery.

Cardiac, renal, epathic, respiratory or metabolic dysfunctions in ASA 3,4,5 can favor a severe diffusive infection, which is potentially life-threatening, and therefore AP is recommended for every invasive dental procedure [34]. In this case, the choice of AP scheme depends on the type of intervention (Figure 1).

AP is also recommended for patients exposed to high-dose irradiation on jawbones and patients receiving intravenous bisphosphonates or denosumab [33] (Table 3). In these patients, the main risk is not strictly related to bacteremia but to bone contamination from invasive procedures that can easily cause osteomyelitis and medication-related osteonecrosis of the jaws (MRONJ) [21,35]. Indeed, amino-bisphosphonate treatment is related to the risk of developing bisphosphonate-related osteonecrosis of the jaws. The prolonged administration of this drug via endovenous infusion or per os (more than 3 years) along with aggravating cofactors (prolonged steroideal/antiangiogenetic therapies, neoplasms) enhances this risk (0.8%–12%) [21]. Osteonecrosis can also be induced by antiresorptive and antiangiogenic drugs, tyrosine kinase inhibitors, mammalian targets of rapamycin inhibitors, selective estrogen receptor modulators (SERMs) and immunosuppressant drugs [36]. Special attention should be paid to cancer patients in bisphosphonate treatments characterized by major potency, dose and duration. In addition, cancer patients may also have other risk cofactors such as a weakened immune systems and/or use of antiangiogenic agent [37].

The most common preceding local event to BRONJ and/or MRONJ is tooth extraction (55.6%) [35]. Irradiated patients present the risk of osteoradionecrosis after tooth extraction in 7% of cases, reduced to 6% when AP is applied [36]. For these patients, AP is recommended in every invasive dental procedure, especially for teeth extractions or bone surgery; when possible, a closure of dental sockets with surgical flaps should be suggested, along with a suspension of pharmacological treatment for 4–6 weeks that is previously agreed on with oncologist [21]. When patients are treated with denosumab, it is recommended to perform surgical procedures within 3 months from the last infusion or within 45 days from the next administration to favor healing of tissues [38]. Although root canal treatments should not be strictly included in bone invasive procedures, the extrusion of infected material in the bone periapical region could induce an inflammatory process; therefore, a short preoperative AP is recommended [33]. While for root canal treatments a classic short preoperative prophylaxis is recommended, for every surgical intervention, especially teeth extractions, a prolonged AP (for more days) based on concomitant use of amoxicilline and metronidazole is strongly recommended, although the length of postsurgical AP is controversial in the literature [21].

As for patients with joint prosthesis, there is little consensus on the link between invasive dental procedures and the risk of developing hematogenous prosthetic joint infections (HPJI). Some investigations did not report evidence that AP reduces the incidence of dental HPJI [39], while the American Academy of Orthopaedic Surgeons and American Dental Association recommend AP for invasive dental procedures executed during the first 3 months after orthopedic intervention, or for patients with joint prosthesis who have previous history of infections or massive oral infection [33].

In general, AP is also recommended in bone surgery with insertion of fixtures (as described below in Section 5.4) and in surgery of selected infected sites [9,12].

## 4. Chlorhexidine

As previously mentioned, risk of postsurgical infectious complications in oral surgery are more related to preoperative and postoperative bacterial contamination than to antibiotic administrations.

Oral hygiene improvement, along with rapid and atraumatic surgery, reduces the incidence of infective complications, especially in exodontic surgery, with the possibility to reduce antibiotics administration. Other adjuvants such as chlorhexidine (CHX) may also be used as antiseptic agents [40]. CHX acts on bacterial membranes and cytoplasmic components inducing cell death [41]. CHX is one of the antiseptic agents more used in dental practice and in other disciplines. Its formulations in dentistry are principally produced in gel and mouthwash with concentrations of 0.12%, 0.2%, 0.50% and 1% [21,39]. There are controversies on which formulation is more effective: while gel formulations seem to have longer and direct action on the surgical site, mouthwash is easier to apply, especially when there are sutures, and/or in extensive surgical procedures with wide or multiple surgical sites. Efficacy of CHX mouthwash is strictly dependent upon the dose and time of exposure [42]. For effective inhibition, mouth rinsing twice daily for 60 s with 10 mL of a 0.2 % solution of CHX is required [25,43], paying attention to accurate removal of dental biofilms that could reduce diffusion and efficacy of CHX [40].

Specifically, in exodontic surgery CHX has proved to be a good prophylactic agent for dry socket in 0.2% gel formulations with two daily applications during the postsurgery period [44]. As for the antiseptic properties in the surgical site, some authors postulated an indirect anti-inflammatory effect with reduction in inflammatory mediators produced as a result of bacterial activity, with a beneficial effect on the perception of postoperative pain [40].

Although CHX should be considered an antiseptic, bacteria can acquire mechanisms of resistance. For this reason, it is important to use effective concentrations, favored by a mechanical debridement of biofilms, avoiding long periods of treatment. One of the main acquired mechanisms of CHX resistance depends on multidrug efflux pumps, transmembrane proteins forming channels that remove toxic substances from the cytoplasm and the cytoplasmic membrane [45].

The efflux pumps are coded by the plasmid-borne *qac* (quaternary ammonium compound) gene family (*qacA, qacB, qacC*), with an induced transcription and upregulation by exposure to CHX [46]. Low levels of exposure to CHX (as in oral biofilms) may result in inefficacy and development of resistance. Furthermore, efflux pumps recognize other antiseptics and antibiotics as substrates so that antiseptic resistance (ASR) may be associated with an acquired cross-resistance to other antibiotics [47]. Some studies also suggest that acquired ASR can induce AMR with unclear mechanisms [48], as CHX-resistant bacteria display resistance to a wide range of antibiotics including betalactams, gentamicin and tetracyclines [42]. The concomitance of AMR and ASR is not only explained inducing multidrug efflux pumps, in actual fact *qac* genes coding for efflux pumps are frequently located on plasmids with various other resistance genes [49]. Induced upregulation of one gene can determine a simultaneous transcription of other genes, developing several AMR mechanisms [42].

## 5. Use of Antibiotics in Dentistry

### 5.1. Antibiotics in Endodontics

Many studies (reviewed in [33]) have shown the inappropriate prescription of antibiotics to treat endodontic infections such as irreversible pulpitis, pulp necrosis and periapical periodontitis. In these cases, a lack of the pulpal bloodstream prevents antibiotics reaching endodontic and periapical regions and the only effective therapies are an endodontic treatment or a tooth extraction. A deep dental decay determines invasion of bacteria nearby pulpal tissue, determining an inflammation that can evolve in pulpitis, characterized by high spontaneous pain. After the complete pulpal necrosis, bacteria colonize the apical root region and their metabolic products determine a chronic inflammation in bone periapical region with immune response clinically recognized by pain induced by percussion/biting and radiographic presence of periapical radiolucency (periapical periodontitis) [50]. To bypass the lack of blood circulation in the root canal, topical antibiotics have been proposed as medicaments during endodontic procedures, but no scientific evidence actually supports this use. Furthermore, the use of topical antibiotics can determine chromatic alterations of dentin [33]. As chemical adjuvants in root canal treatment, sodium hypochlorite, chlorhexidine and ethylene diamine tetraacetic acid (EDTA) are recommended [51]; calcium hydroxide or meta-cresil-acetate may be used as intermediate medicaments between appointments for their antiseptic properties [52].

**Figure 3:**
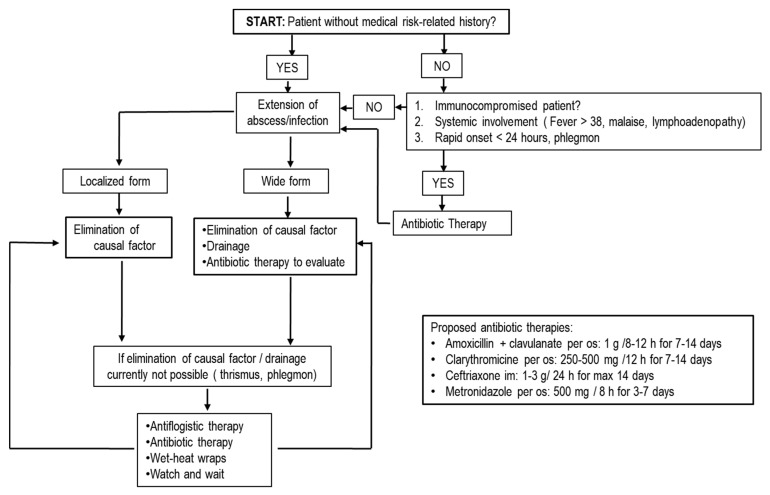
Flowchart for abscesses/suppurative infections in adult patients. In children, posology adjustments must be used as described in Table 1 and Table 2. Abbreviations: g = grams; h = hours; per os = oral administration; im = intramuscularly.

In borderline cases of teeth with pulpal exposure, it is possible to maintain pulpal vitality avoiding endodontic treatment and adopting pulp capping. Additionally, in these cases, the use of topical antibiotics is not supported by evidence of efficacy [33] and several alternative materials, such as mineral trioxide aggregate, calcium hydroxide and adhesive systems, are generally preferred [53]. Management of traumatic injuries of teeth is linked to endodontics and conservative dentistry. Traumatic accidents can determine fractures, luxations or avulsion of tooth as possible injuries. Tooth fractures can be managed with reconstructive procedures with possibility of endodontic treatment and, as previously described, they do not require systemic antibiotics [33]. AT is necessary in luxations and avulsion reposition or replantation of tooth with its mechanical blocking during healing phase of root on alveolar bone [33]. During the healing phase, bacterial contamination can compromise prognosis with an inflammatory root resorption; AT or a topical application of antibiotics, such as tetracyclines on root surface, offer an effective advantage in periodontal and pulpal healing only in replantation of avulsed teeth [54]. Current positions in conservative dentistry and endodontics identify a minority of diseases and treatments where systemic or a topical AT is recommended, including acute infective diseases such as alveolar acute abscesses and postsurgical infections [12,15,33,50] (Figure 2 and Figure 3).

A postsurgical infection can be evaluated by various signs/symptoms: suppuration after 72 h from surgery, spontaneous or induced pain and swelling persisting after 48 h, lymphadenopathy, local tension of tissues and fever over 38 °C, malaise and trismus [55]. Clinical attention must be focused on pain or swelling that may not necessarily be caused by infection but could be a consequence of a surgical trauma. Managing orofacial pains and swellings with AT without a clear diagnosis of infection is useless and harmful [56]—for instance, in the case of pericoronitis, an inflammation of soft tissues covering crown of impacted tooth, especially in mandibular third molars [57]. Pain, swelling and trismus are common clinical manifestations that can be managed with antiphlogistic and antiseptic therapies, followed by tooth extraction and AT, and should be prescribed only in presence of specific infective complications [58].

An abscess is defined as a neo-formed cavity with pus accumulation subsequent to infection and the clinical symptoms are generally a fluctuant swelling with tension of involved tissues and spontaneous pain. Acute abscesses can have an endodontic cause, starting from a deep decay with bacterial invasion of pulpal space and diffusion in periapical bone and subsequently in soft tissues. Abscesses may originate from a deep and obliterated periodontal pocket where suppuration does not have spontaneous drainage [33].

The first-line strategy in treatment of acute abscesses is the elimination of the causal factor (endodontic infection, deep pocket, impacted tooth) with conservative treatment or tooth extraction instead of AT. This can be sufficient in localized forms (localized fluctuant swelling delimitated in alveolar mucosa). In wide forms, where infection and subsequent suppuration involve and diffuse subcutaneously between fascial spaces (submandibular space, cheek space, neck fascial space), drainage with elimination of suppurative content can be necessary [59]. Drainage allows rapid and massive removal of toxic products, decompression of fascial spaces and better penetration in tissues of eventual AT [33].

AT can be necessary in progressive infections with rapid onset (less than 24 h) and when drainage/or elimination of causal factors (phlegmon or trismus) is not feasible. It is also necessary in alveolar abscesses with systemic involvement (fever over 38 °C, lymphadenopathy, malaise) and in alveolar abscesses of immunocompromised patients [33] (Figure 3).

In order to focus on the importance of the elimination of causal factors and execution of drainage, several authors report that all categories of antibiotics used after these procedures are equally effective [17].

### 5.2. Antibiotics in Periodontology

First-line treatment of periodontal diseases is the mechanical elimination of bacterial biofilms on the tooth surface and improvement of oral hygiene with adequate brushing techniques. AT can be added only for serious types of periodontal diseases such as necrotizing forms, early insurgence periodontitis, periodontal abscesses and nonresponsive forms [60]. Usage of AT in combination with scaling and root planing showed a modest additional probing pocket depth (PPD) reduction in comparison with scaling and root planing alone [61]. With regard to the antibiotic type, meta-analyses showed that only doxycycline and the combination of amoxicillin with metronidazole resulted in significant PPD reduction. Systemic AT schemes suggested by scientific societies with major consensus are metronidazole plus amoxicillin or tetracycline (250 mg four times daily for 2 weeks) [62].

Another therapeutic strategy in combination with mechanical debridement is the use of topical antibiotics applied in periodontal pockets. A recent systematic review has shown that local antimicrobials are effective in reducing probing pocket depth (PPD) and increasing the clinical attachment level (CAL) in diabetic patients [63].

The most studied drugs are tetracyclines (tetracycline hydrochloride, doxycycline and minocycline), broad-spectrum molecules capable of inhibiting collagenases, metalloproteinases and interleukins [64]. Topical tetracyclines are vehiculated in periodontal pockets with etylenvilacetate fibers or in gel formulation. Moreover, topical AT with tetracyclines in combination with nonsurgical periodontal treatments enhance PPD reduction with respect to mechanical biofilm removal alone [65]. Additionally, metronidazole can be used in gel formulation with a 25% concentration. Metronidazole gel does not seem to achieve significant benefits with respect to nonsurgical treatments alone [66]. Considering the risks of developing AMR and the actual controversial results in the literature on the real clinical benefits of a combined AT, antibiotics should be used only in selected and serious types of periodontal diseases, such as necrotizing forms, early insurgence periodontitis, periodontal abscesses and nonresponsive forms and in every case only in combination to periodontal treatments to break up and remove bacterial biofilms. Systemic AT can be adopted for wide periodontitis with several deep periodontal pockets that are nonresponsive to mechanical treatment and in acute periodontal abscesses. As previously described for treatment of acute alveolar abscesses, systemic AT in periodontal abscesses should be accompanied by elimination of the causal factor and/or drainage execution. The first-line antibiotic in periodontal abscesses treatment is metronidazole, used alone (500 mg metronidazole three times daily for 3–7 days) or in combination with betalactams (500 mg amoxicillin + 500 mg metronidazole three times daily for 3–7 days) [21]. Topical AT can be selected in localized forms with a few periodontal pockets to improve antibiotic concentration in situ.

### 5.3. Antibiotics in Extractive Oral Surgery

Postoperative complications of dental extractions can be various. With exception of hemorrhagic and neurological complications, mainly related to patients’ conditions and/or technical difficulties—the most common infective complication is the dry socket (alveolar osteitis). Dry socket (DS) is unproperly considered an infection, as it is a delayed healing process with an hypothetical infective concause [67].

Postsurgical pain, a common symptom of DS, and swelling are not necessarily related to postsurgical infection, but could indicate a stronger postsurgical inflammatory response. To diagnose a postsurgical infection and the necessity of AT, postsurgical pain and swelling should persist after 48 h and this should be accompanied by other signs and symptoms such as suppuration 72 h after surgery, lymphadenopathy, local tension of tissues, fever over 38 °C, malaise and trismus [68]. Indeed, teeth extractions can have various levels of technical difficulty depending on position of teeth in dental arch, root anatomy (long or curved), need of elevation of surgical flaps, osteotomy and odontotomy. High levels of difficulty are related to an increase in surgery time and surgical trauma, with consequent increase in inflammatory response [56,69]. In addition to the technical difficulties, in order to predict odds ratios of possible complications, age of patient, systemic conditions, and bacterial load must be considered [67]. Systematic antibiotic prescription for all types of extractions can represent a treatment without clinical justification, exposing patients to risks of adverse effects and promoting AMR [1]. Pain and swelling appear to be more related to surgical trauma (elevation of surgical flaps, osteotomy and traumatic extraction) with a long surgery time and age of patient, than to antibiotic administrations [69].

Local infections and DS are strictly associated with bacterial load pre- and postintervention. Indeed, extractions of teeth with periodontal infections have a high probability of developing dry socket (odds ratio of 7.5) caused by bacteria spreading in socket bone [45]. Additionally, smoking can cause a high rate of postsurgical local infections [70]. By practicing a correct preoperative disinfection of the operating field, with elimination of bacterial biofilms and tartar, and maintaining a low bacterial load in the postoperative healing phase, it is possible to reduce the onset of an inflammatory response and incidence of potential local infections, avoiding AT [11,23].

The etiology of DS is increased fibrinolytic activity that disaggregates blood clots as a result of plasminogen pathway activation. This can be achieved by chemical mediators released by surgical trauma, or secreted by bacteria (such as streptochinases) [67]. Surgical trauma, surgical time and bacterial load should be considered as the main risk factors. Excessive bone surgical trauma could determine injury of the bone lining of the socket and thrombosis in underlying vessels, resulting in a delayed healing phase, with predisposition to infection; moreover, a long surgical time can enhance risks of bacterial contamination and bone exposure [70]. Haraji and Rakhshan described an increase in DS incidence with age, probably due to the slower metabolism, poor tissue quality and weaker immune system [40]. Additionally, teeth with recurrent infections are more prone to developing DS [40]. Improving oral hygiene and preoperative mechanical removal of dental biofilms are good practices for decreasing incidence of DS and antiseptic agents can contribute to maintain a low bacterial load: chlorhexidine rinse or gel used pre- and postextraction may reduce the incidence of DS. Preprocedural mouth rinse with 1% povidone or 1% peroxide hydrogen can also be valid adjuvants to reduce bacterial load [71,72].

A reduction in DS incidence can be obtained by correct socket management with cold irrigation of the postextraction socket to remove bacteria or debris and adequate scraping of alveolar bone plates to remove granulation tissue, bacteria and to favor osseus bleeding. In patients with dysfunction of coagulation where blood clot is more susceptible to fibrinolysis, the use of sutures and absorbable collagen sponges to improve blood clot stabilization and platelet-rich plasma that enhances healing speed appear to be useful. Some authors suggest AP to reduce bacterial production of streptochinases, but no randomized trials have been carried out [70]. Antibiotic prescription in every possible regimen is not very effective in terms of preventing dry socket [1]. A low reduction in dry socket incidence in third molar surgery is described for preoperative AP, with the possibility of avoiding one case of DS in every 13 patients by prescribing antibiotics. The risk/benefit ratio can be carefully evaluated in order to increase the AMR to prevent complications [56,69]. A correct management of bacterial load with full-mouth disinfection and an atraumatic surgical technique should be preferred. Antibiotics are not effective for treatment of dry socket. Irrigations with saline solution to remove bacteria and necrotic debris and surgical curettage of alveolar bone plates to promote a new osseus bleeding with neoformation of a new blood clot are the suggested procedures [70]. Accordingly, the majority of authors recommend avoiding the use of antibiotics in routine dental extractions that do not require osteotomy or surgical flaps in healthy individuals [1]. No statistically significant difference in postoperative complications (pain, fever, swelling, infection and DS) was found among groups that received antibiotics in every therapeutic scheme (preoperatively and/or postoperatively) and the control group [1]. Conversely, in complex extractions treated with antibiotics (surgery of long duration, surgical flaps with osteotomy), there was a reduction (from 2.7% to 16%) in postsurgical infections (suppuration, pain, swelling, trismus, dry socket, pyrexia, trismus, heat). Correct preoperative planification should help predict difficult levels of extraction and assess the corresponding infection risk. The decisional checkpoints to consider are: (i) the need of osteotomy and related bone exposure; (ii) osseus surgical trauma and long duration of surgery with prolonged bone exposure. All these conditions can favor bacterial contamination [56].

The need to elevate surgical flaps can determine soft tissue trauma, especially with excessive traumatisms or incorrect sutural positioning, favoring infection or other complications. For extractions with expected long durations, preoperative AP is recommended, whilst postoperative AP in the case of osteotomy is not necessary. In these cases, the use of antibiotics reduces the risk of infection, but it is poorly related to other signs/symptoms related to surgical trauma (pain, swelling, trismus). These symptoms can be suggestive of an inflammatory response to surgical trauma or even be the result of trauma and concomitant infection [69].

Although there is little evidence that use of antibiotics for third molar extractive surgery requiring bone removal in healthy young adults can reduce incidence of infection and DS, the odds ratio is one person for every 12 to 17 patients given antibiotic prophylaxis. In comparison, for adverse effects associated with antibiotic administration, the odds ratio is one adverse effect for every 21 persons treated with antibiotics, with further risk of inducing antibiotic resistance. No significant differences were observed between antibiotics and placebo in terms of fever, swelling, trismus and pain [56]. It is yet unclear if preoperative prophylaxis is equally effective to AP prolonged for more days postoperatively. For resective periodontal surgery with osteotomy, the same prolonged AP is suggested, whereas for mucogingival surgery, AP is recommended only in compromised patients, focusing the attention more on periodontal health, to have a preoperative field with low bacterial load, and focusing on correct management of soft tissues to reduce surgical trauma [60] (Figure 1).

### 5.4. Antibiotics in Implantology and Regenerative Techniques

Bacterial contamination can determine colonization of bone substitutes, bone grafts and barrier membranes used in regenerative techniques or surface implants [11]. Due to the lack of blood vessels, these biomaterials provide a protected substrate for bacterial colonies, hindering their elimination and resulting in persistent inflammation [73]. Although a physiological inflammation is necessary to initiate the repair cascade for tissue healing, a persistent inflammatory response interferes with the process of healing and regeneration of soft and hard tissues [74]. An infected site interferes with bone formation in some inflammatory systemic diseases such as uncontrolled diabetes [75] or rheumatoid arthritis [76], characterized by an exaggerated inflammatory response that may become chronic, resulting in interference in the healing process of soft and hard tissues [74]. Traditional guidelines and empirical evidence have always associated these types of surgery with prolonged antibiotic protocols, generally starting preoperatively to continue some days after surgery, with the belief that it is necessary to improve success rates and osteointegration, and to reduce the incidence of postsurgical infections [77].

Implant failure is essentially defined as a mobile implant and is not necessarily related to previous or clinical presence infections. Failures can be classified as early failures, occurring prior to prosthetic restoration and generally caused by biological factors (bacterial contamination/infection, low osteointegration, healing disorders), or late failures after prosthesis placement where mechanical factors (occlusal overload) may overlap with biological factors (peri-implantitis) [78]. Postoperative infections after implant insertion can present suppuration, sinus tracts, spontaneous pain, swelling, local tension and pain of soft tissues and mucosal erythema, generally protracted until 8 weeks after surgery but do not necessarily determine implant failure [79].

Currently, the use of antibiotics to prevent implant failure or postsurgical infection in dental implants is highly controversial [80] and postoperative antibiotics seem to be ineffective in implant success rate [81]. While AP in implant surgery is recommended for the categories of patients previously described (i.e., patients with high risk of infection and/or infective endocarditis), for other patients, a single preoperative dose of 1, 2, or 3 g of oral amoxicillin 1 h prior to implant placement may be effective in preventing and reducing dental implant failures, but no significant effects were observed with regard to occurrence of postoperative infections [78]. Another antibiotic scheme used in implant and bone regenerative procedures is 2 g amoxicillin as a single dose 1 hr before intervention and 500 mg of amoxicillin three times daily (8-hourly) for 1–3 days [56].

Although several authors describe the use of oral amoxicillin as postoperative AP, even associated with preoperative regimen, this treatment is ineffective/poorly evidenced for the reduction in implant failures and for prevention of postoperative infections [82]. Although AP seems to be effective, the real effectiveness of preoperative AP in reduction in risks of implant failure is approximately of 1.3%–2%, with a ratio of one implant failure prevented for 25–77 implant insertions with preoperative AP, coupled with the controversial questioning on ratio risks/benefits regarding the onset of AMR [82]. As for bone regenerative techniques, systemic antibiotics do not seem to provide any improvement in postsurgical complications such as pain, swelling, hematoma and bleeding, flap closure and wound healing, suppuration and implant stability in healthy patients with periodontal health [11].

Medical reviews recommend high standards of reduction in bacterial contamination load pre-, peri- and postsurgery, eliminating infective foci and reaching periodontal health before implant insertion or regenerative procedures [83]. More so than antibiotics administration, uncontrolled periodontititis may represent a risk of failure and infection for implants of 4–14 major in relation to periodontal health patients. Several authors suggest that persistent pockets may act as bacterial reservoirs, thereby facilitating microbial colonization of medical devices inserted in bone and determining a possible postsurgical infection and/or surgical failure [82].

Persistence of dental biofilms, oral foci, and postoperative poor oral hygiene can enhance bacterial contamination and an aggressive or incorrect technique on bone (poor irrigation, high drilling speed, hard density bone, drills with reduced cutting quality) or on soft tissues (inadequate incision, traumatic elevation and manipulation of surgical flap, sutures with excessive tension) can determine an excessive inflammation and a suitable substrate for bacterial colonization [11].

Even in ideal conditions with a low bacterial load and healthy periodontal status, bacterial contamination is possible and can be triggered by contact of the surgical bone site and/or implant surface with contaminated saliva, even if it should be rapidly cleared by the immune response [74]. When bacterial infection is not cleared, the contamination can evolve into persistent subclinical infection, with unclear mild symptoms and alterations of the healing phase, with a reduction in final bone implant contact and eventually early implant failure. If contamination is more important and/or favored by surgical factors or patient’s conditions, it can become a clinical infection (postoperative infection) with the same interference in healing and early failure [78,79].

As for the importance of a low bacterial load and atraumatic techniques, some investigators believe that a prolonged AP is useless. Additionally, accepting the hypothetical efficacy of antibiotics to prevent postsurgical infections, in presence of a high bacterial load, the limited period of AP (7–14 days maximum) seems to be insufficient to manage possible contaminations due to the long healing times of bone regeneration and osteointegration. Additionally, excessive surgical traumatisms, with highly related inflammations, might not be effectively managed with antibiotic/antiphlogistic therapies.

Short preoperative AP appears effective in reducing implant failure rates probably because in critical moments of intrasurgery contamination there is an adequate concentration in blood/fluids that can reach the surgical site and implant surface, contributing to a rapid eradication of bacteria [10,78]. However, AP may not be sufficient in case of high bacterial loads or excessive traumatisms of tissues.

## 6. Conclusions

As AMR is perceived as a health emergency, evaluation of the real efficacy of antibiotic prescriptions in dental practice is a very important and high-priority topic. Moreover, restricting antibiotic prescriptions only to clinical situations where they are really effective may determine minor incidence of adverse pharmacological effects and reduce sanitary expenses. Indeed, use of drugs without effective benefits can only determine risk of adverse effects and be a waste of money.

Current guidelines recommend antibiotics for management of suppurative orofacial infections only when elimination of causal factors and drainage are not sufficient, or for systemic involvement, focusing on antibiotics merely as adjuvants. In endodontics, antibiotics must be considered only as AP for specific patients to avoid risks of potential bacteremia, without any effects on the success rate of therapies or any improvement of clinical signs/symptoms. The same guidelines can be recommended in oral surgery, where routine tooth extractions in healthy patients can be managed without antibiotics. Choice of AP can be made for compromised patients (immunocompromised, high risk of endocarditis, high risk of osteonecrosis) and for high-difficulty extractions such as lower wisdom teeth, with the possibility to establish a preoperative scheme or to prolong the treatment for few days after intervention. Crucial checkpoints must be the surgery time and the need for osteotomy, which are usually related to bacterial contamination and surgical trauma. In implantology and regenerative procedures, oral surgery must keep in mind bacterial load and atraumatic surgery to decrease the risks of contamination and failures. Periodontally health patients with low bacterial loads can potentially be treated with a short AP before implant and/or regenerative procedures. To reduce bacterial load, antiseptic agents such as chlorhexidine are useful for short periods and with the correct prescription. These recommendations elucidate the need to reduce antibiotic consumption without reducing clinical success; the aim is to decrease AMR and the life-threatening consequences of AMR and to encourage the idea of personalized medicine in dentistry, a tailored therapy based on patients’ needs, in order to improve the efficacy of therapy and reduce the adverse effects.

## 7. Highlights

➢ Antibiotics in dental practice can only be used in selected situations, for specific patients and using appropriate techniques, generally with short preoperative schemes. Postoperative schemes can be adopted for long-duration surgeries with osteotomy or for antibiotic therapies in complicated abscesses.
➢ Antibiotics must be considered as pharmacological adjuvants that cannot cover or replace medical intervention.
➢ Correct management of oral bacterial load/contamination with elimination of infective foci, dental biofilms and good periodontal health, along with atraumatic surgical techniques, are the main factors influencing the success rates of interventions, rather than antibiotic administration.
➢ Antibiotics are not able to reduce clinical symptoms such as pain and swelling.
➢ Routine tooth extractions in healthy patient can be executed without antibiotics, with the same incidence of complications.
➢ Implant insertion can be managed with short preoperative AP to reduce risk of failure, whilst no beneficial effects are obtained with postoperative regimen schemes.
➢ Chlorhexidine must be used properly and for short periods due to the possibility of inducing cross-resistance to antibiotics.

## Figures and Tables

**Table 1:** Prolonged antibiotic schemes [11,12,14,15,17,18,19,20,21].

First Choice
Amoxicillin = 1 g with oral administration (per os)/8 h for 7 days or more
Amoxicillin + clavunalate = 1 g per os/8–12 h for 7 days or more
Amoxicillin (500 mg per os/8 h) + metronidazole (500 mg per os/ 8 h) = AP for patients at risk of bisphosphonate-related osteonecrosis of the jaws (BRONJ) or medication-related osteonecrosis of the jaws (MRONJ); AT in combination with nonsurgical treatment for aggressive periodontitis
**Patients Allergic to Betalactams**
Claritromicine= 250–500 mg per os/12 h for 7–14 days
Azitromycine= 500 mg per os/24 h for 3 days or more Clindamycin= 300 mg/6 h for 7–14 days
**Posological Adjustments in Children (Weight < 20 Kg and/or Age < 10 Years)**
Amoxicillin = 12.5–25 mg/Kg/8 h
Macrolids = 15 mg/Kg/24 h Clindamycin = 5 mg/Kg/6 h

**Table 2:** Short preoperative antibiotic prophylaxis [13,14,15,20].

First Choice
Amoxicillin = 2 g with oral administration (per os)/1 h before procedure
Amoxicillin + clavunalate = 2 g per os/1 h before procedure
**Patients Allergic to Betalactams**
Macrolides = 500 mg per os/1 h before procedure
Clindamycin = 600 mg per os/1 h before procedure
**Posology Adjustments in Children (Weight < 20 Kg and/or Age < 10 Years)**
Amoxicillin = 50 mg/Kg
Macrolides = 15 mg/Kg Clindamycin = 20 mg/Kg

**Table 3:** Categories of patients where antibiotic prophilaxis is recommended.

● Patients at high risk of infective endocarditis
● Immunocompromised patients with leukopenia <3.500 u/mm^3^ or seral levels of immunoglobulins <2 g/L
● Patients ASA 3,4,5
● Patients undergoing to high dose irradiation on jawbones, or to assumption of amino-bisphosphonates/denosumab
● Patients with joint prosthesis with high risk of adverse outcomes
● Patients undergoing prolonged and extensive surgical interventions
● Patients undergoing surgery in infected sites
● Patients undergoing insertion of fixtures and/or biomaterials

Abbreviations: American Society of Anestesiology (ASA).

## Data Availability

Not applicable
